# UiO-66 with Both Brønsted and Lewis Acid Sites for Catalytic Synthesis of Biodiesel

**DOI:** 10.3390/molecules29174195

**Published:** 2024-09-04

**Authors:** Yu Wang, Zhimin Yang, Xichang Wu, Wenxuan Quan, Qi Chen, Anping Wang

**Affiliations:** 1 Key Laboratory for Information System of Mountainous Area and Protection of Ecological Environment of Guizhou Province, Guizhou Normal University, Guiyang 550025, China; wangyu@gznu.edu.cn (Y.W.);; 2 School of Materials and Architectural Engineering, Guizhou Normal University, Guiyang 550025, China; qichen@gznu.edu.cn

**Keywords:** metal–organic frameworks, biodiesel, sulfonation, active sites

## Abstract

In the present study, an acid catalyst (UiO-66-SO_3_H) with Brønsted and Lewis acid sites was synthesised for the preparation of highly efficient biodiesel from oleic acid and methanol using chlorosulphonic acid sulfonated metal–organic frameworks (UiO-66) prepared with acetic acid as a moderator. The prepared catalysts were characterised using XRD, SEM, FT-IR and BET. The catalytic efficiency of the sulfonated catalysts was significantly improved and successful sulfonation was demonstrated by characterisation techniques. Biodiesel was synthesised by the one-pot method and an 85.0% biodiesel yield was achieved under optimum conditions of the reaction. The esterification reaction was determined to be consistent with a proposed primary reaction and the kinetics of the reaction was investigated. A reusability study of the catalyst (UiO-66-SO_3_H) was also carried out with good reproducibility. In conclusion, the present study provides some ideas for the synthesis of catalysts with high catalytic activity for the application in the catalytic preparation of biodiesel.

## 1. Introduction

With the development of scientific and technological progress and fast industrialisation, the demand for some traditional energy sources has been increasing, which has resulted in the over-consumption of some non-renewable natural resources including coal, petroleum and fossil fuels [1]. The global population is expected to increase by 50 percent by 2040, leading to an increasing energy demand, and China’s energy consumption is very high compared to other countries [2]. As the world’s population grows exponentially, the demand for resources continues to increase alongside the rapid decrease of natural resources and the acceleration of the global environmental crisis. Researchers are therefore urgently looking for new, more sustainable and environmentally friendly energy sources to replace traditional fossil energy sources [3].

**Figure 1:**
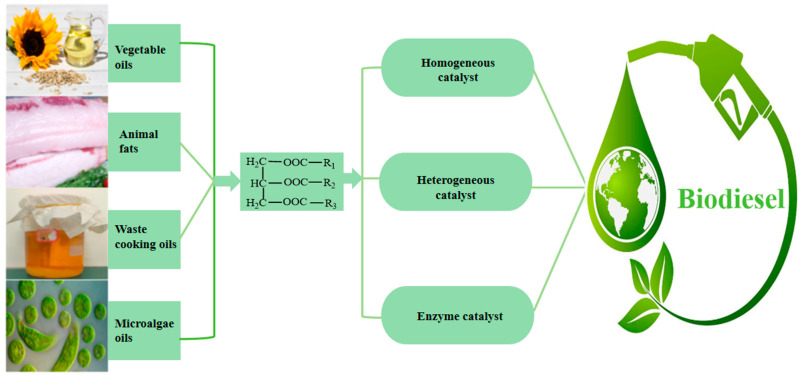
Feedstock for the preparation of biodiesel.

Biodiesel is a green, non-toxic, renewable liquid biofuel. Biodiesel is virtually free of sulphur and aromatic compounds and is biodegradable, making it truly environmentally friendly [4], and it also has similar physicochemical properties to petroleum and fossil fuels [5]. Biodiesel is widely exploited due to its many environmentally friendly properties, such as its green and sustainable regeneration, and it is also a source of raw materials and intermediates for many chemical products, such as some surfactants [6] and lubricants [7]. Biodiesel is derived from vegetable oils, animal oils, waste cooking oils, microbial fats and oils as shown in Figure 1.

Biodiesel can be produced by esterification and transesterification of natural oils and fats with alcohols such as methanol and ethanol [8]. The esterification of alcohols with natural fats and oils produces esters, and a certain amount of catalyst is required for the production of esters, but there are many types of catalysts, including homogeneous catalysts, non-homogeneous catalysts and enzyme-activated catalysts. Homogeneous catalysts including NaOH, KOH, HCl and H_2_SO_4_, are used in the transesterification of vegetable oils and animal oils with a low acid value as feedstock for the conventional preparation of biodiesel due to their high reactivity and low cost. Due to its difficult recovery, dangerous operation process, low ability to be separated from the product, high corrosiveness to the equipment, high requirements on raw materials and reaction conditions, and ease with which it causes saponification reaction, the catalyst can easily lose its activity, which are the obstacles that led to its development in the industrial process [9]. For enzyme catalysts, the synthesis process is characterised by low environmental pollution, high purity, mild reaction conditions and few by-products. However, the high cost of production, lower reusability and lower stability limit their use in many fields [10]. Non-homogeneous catalysts such as modified biochar, zeolites and metal-organic frameworks are suitable for the catalytic preparation of biodiesel from high acid value oils and fats. These catalysts have the advantages of easy separation from the product, high reusability, high stability, being less prone to saponification, being easy to recycle, high selectivity and high catalytic activity [11,12]. Therefore, the synthesis of non-homogeneous catalysts with high specific surface area and large pore size with many active sites is one of the most urgent problems to solve [13,14].

An emerging special material is currently attracting the attention of researchers: metal-organic frameworks (MOFs). Metal-organic frameworks (MOFs) are crystalline materials with multiple clusters of porous properties formed by metal ions and organic ligands, with excellent physicochemical properties, high specific surface area, high porosity, adjustable pore size and a large number of metal-open sites [15,16,17]. Therefore, they are sought after by researchers in sensing [18], biomedicine [19], catalysis [20], environmental remediation [21] and many other fields. Especially in the field of catalysis, they have a pivotal position; various metal-organic frameworks (MOFs) in the catalytic preparation of biodiesel have good catalytic effect. For example, Synthesis of ILe@Cu@MOF mesoporous catalysts applied to the preparation of high-quality biodiesel from Xanthoceras sorbifolia bunge oil [22], PSH/UiO-66-NO_2_ catalysed biodiesel production from jatropha oil [23] and magnetic bifunctional SrO-ZnO/MOF catalysts catalysed the conversion of high-acid- value oil production to biodiesel [24]. Since metal-organic frameworks (MOFs) can be synthesised from different metal ions and different organic ligands, there is some variation in all aspects of their structure and physicochemical properties. Some of the synthetic parameters in the preparation of metal–organic ligands have been revealed over the past two decades, so the primary consideration is to choose metals such as potassium, zirconium, magnesium, iron, etc., which have high selectivity, high efficiency, stability and low toxicity to the environment [25,26]. UiO-66 is one of the Zr-based MOFs. UiO-66 is an octahedron with excellent thermal and chemical stability and catalytic activity, as well as high porosity and a large specific surface area (about 1200 m^2^/g) [27,28,29]. The metal-unsaturated coordination structure in the UiO-66(Zr) structure with 12 ligands concurrently attached can provide abundant Lewis acid sites [30,31]. In order to obtain higher catalytic activity, sulfonation can be carried out by in situ synthesis, with the sulfonic acid group providing a strong Bønsted acid site [32]. Synthesis of MOF materials using acetic acid as a moderator increases the UiO-66 pore volume and accelerates the proton conduction rate, which can be achieved by sulfonation and the addition of acetic acid as a moderator to enable UiO-66 to have both a large pore volume and high acid site density [33].

In the present work a simple one-pot method was used to prepare a solid acid catalyst (UiO-66-SO_3_H) modified with sulfonation groups. Modified with sulfonation groups, the solid acid catalyst (UiO-66-SO_3_H) was rich in Lewis acid sites and Brønsted acid sites, giving the catalyst an abundance of active sites for the preparation of biodiesel by esterification with oleic acid. A range of scientific techniques was employed to analyse the physical and chemical properties of the catalyst’s characterised morphology, to optimise the reaction parameters of the reaction by a detailed one-factor method, and to further explore the reusability and applicability of the catalyst. The kinetics of the esterification reaction and possible reaction mechanisms are also explored in depth. This easy, fast method provides a reference for the synthesis of non-homogeneous catalysts and more directions for the efficient synthesis of biodiesel.

## 2. Results and Discussion

### 2.1. Catalysts Characterisation

**Figure 2:**
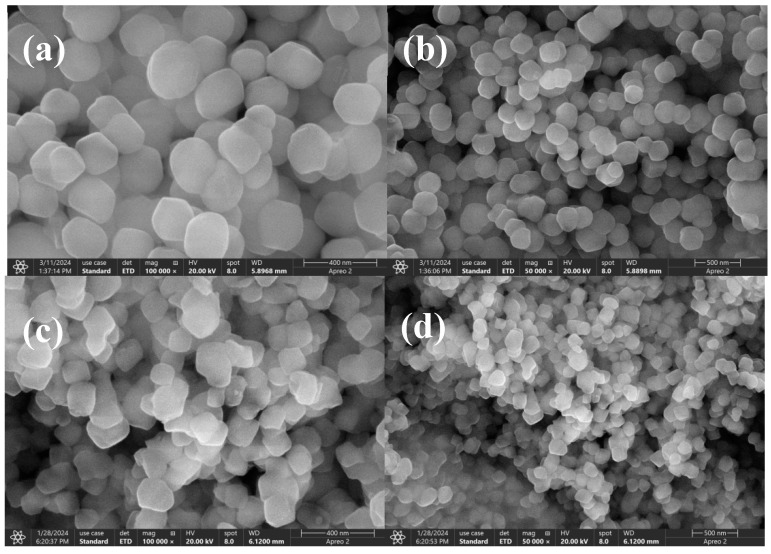
SEM images of: UiO-66(**a**,**b**), UiO-66-SO_3_H (**c**,**d**).

The UiO-66 morphology were seen as a regular octahedral structure by micrographs under SEM [34]. The morphology of UiO-66 and UiO-66-SO_3_H were examined by SEM as shown in Figure 2. The characterisation map of UiO-66 had a porous regular octahedral structure as in Figure 2a,b. The chlorosulfonic acid sulfonated modified UiO-66 had a good agglomeration effect, and the arrangement was more compact as shown in Figure 2c,d. The surface of UiO-66-SO_3_H was porous, but the pore sizes were not uniform, which may be due to the collapse of the surface structure during the drying process [35].

**Figure 3:**
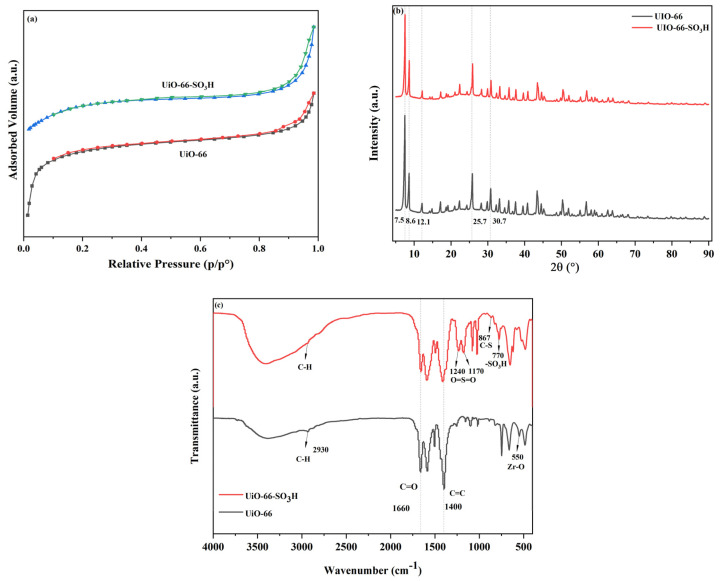
(**a**) shows the N_2_ adsorption–desorption curve, (**b**) shows the XRD spectrum and (**c**) shows the FT−IR spectrum.

The N_2_ adsorption–desorption curves of UiO-66 and UiO-66-SO_3_H are displayed in Figure 3a. According to the International Union of Pure and Applied Chemistry (IUPAC) classification, the curves in Figure 3a all belong to type I mesoporous materials, and their specific surface areas and average particle sizes are shown in Table 1. The decrease in the specific surface area of UiO-66-SO_3_H relative to UiO-66 after UiO-66 has been sulfonated may be due to the large volume of -SO_3_H groups on the framework of UiO-66-SO_3_H, which led to an increase in the specific gravity of the unit skeleton. It is also possible that the presence of a large number of -SO_3_H groups may have caused some clogging of the pores. It is also possible that the pore blockage may have been caused due to the site resistance effect of -SO_3_H. These results are also in agreement with the related literature reports [36].

The XRD patterns of the catalysts are shown in Figure 3b. It can be seen that the synthesised crystalline materials show characteristic peaks at 2θ = 7.5°, 8.6°, 12.1°, 25.7° and 30.7°, which are consistent with the positions of the simulated UiO-66 standard peaks as well as with the (111), (200), (022), (113) and (224) crystalline surfaces of UiO-66 [32]. They indicate that the sulfonated catalysts maintained the crystalline structure of the parent UiO-66, respectively, and also proves the successful synthesis of the sulfonated catalysts. Successful grafting of the sulfonated groups showed that the sulfonating agent was uniformly distributed and well-stabilised in the pristine UiO-66 [37].

The FT-IR spectrograms of the two MOF materials are illustrated in Figure 3c. In order to prove whether the sulfonated groups were grafted into the MOF framework, it can be seen from the figure that the peaks at 2930 cm^−1^, 1660 cm^−1^,and 1400 cm^−1^ are the C-H, C=O and C=C in the aromatic moiety from the stretching vibration of the organic ligand, respectively [38]. A typical telescopic vibrational peak belonging to Zr-O appeared at 550 cm^−1^ [39]. The feature peaks appeared at 1240 cm^−1^. The measurement of 1170 cm^−1^ belonged to the O=S=O the tensile oscillation. This is also consistent with what was previously reported by Luo et al. [40], where 867 cm^−1^ belonged to the C-S stretching vibration [41]. The sulfonated group -SO_3_H appeared as a characteristic peak at 770 cm^−1^ [42]. The successful grafting of the sulfonated groups was evidenced by the conjugation effect and electronegativity of the sulfonated groups, which resulted in a difference in the absorption bands of the two peaks.

### 2.2. Biodiesel Yield Analysis

**Figure 4:**
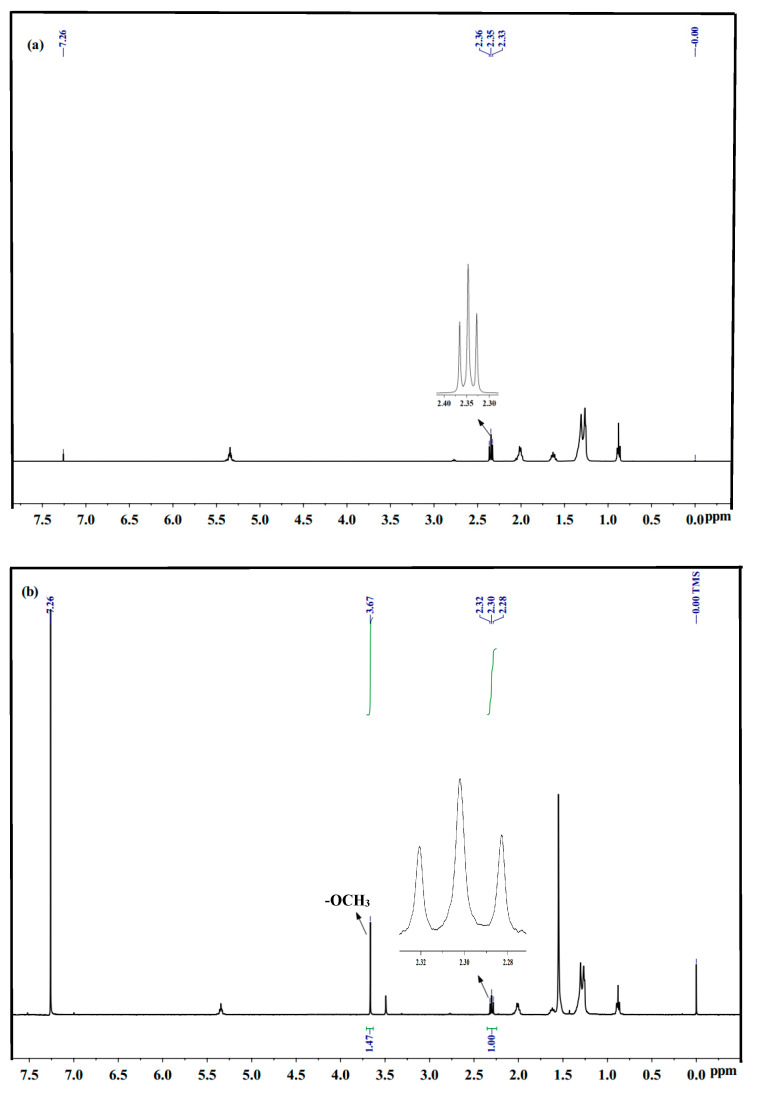
(**a**) represents the hydrogen spectrum of oleic acid, (**b**) represents the hydrogen spectrum of biodiesel.

Nuclear Magnetic Resonance Hydrogen Spectroscopy (NMRHS) is a rapid method to detect the methyl oleate biodiesel yield [43]. Tetramethylsilane (TMS) was used as the internal standard and CDCl_3_ as the solvent during the test. The calculations were based on the difference in the displacement of the hydrogen peaks in the substrate and product before and after the esterification reaction. The ^1^H-NMR signatures of oleic acid and methyl oleate were essentially similar as seen in Figure 4a,b. After the end of the catalytic reaction there was a particularly clear difference, with a new single peak for methyl oleate at 3.67 ppm, i.e., -OCH_3_ post-esterification, and an essentially unchanged triplet of peaks at about 2.30 ppm prior to and after reaction, which is typical of the α-CH_2_ peak. The yield of methyl oleate can be determined from the peak areas of α-CH_2_ and -OCH_3_. The below Equation (1) was used to assess the yield of methyl oleate. The yield of methyl oleate was calculated as 98.0% from the cumulative peak area in Figure 4b, and as 86.6% from acid-base titration under the same conditions, and there was not much difference in the yield of methyl oleate between the two calculations.

(1) $$∁=100\times \left(\frac{{2A}_{Me}}{{3A}_{CH2}}\right)$$

$A_{Me}$—Peak area of -OCH_3_

$A_{CH2}$—Peak area of α-CH_2_

### 2.3. Optimisation of Reaction Conditions

**Figure 5:**
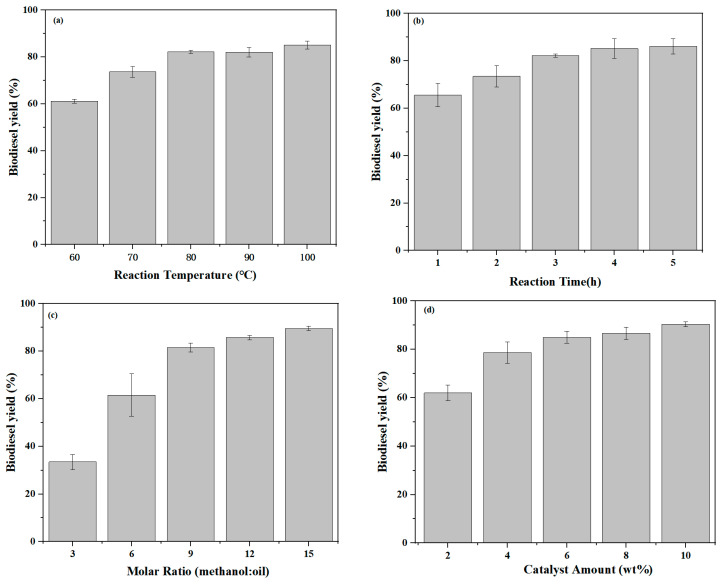
Effect of esterification parameters on biodiesel yield. (**a**) Reaction temperature (t = 3 h, alcohol–oil molar ratio of 9:1, catalyst dosage of 6 wt%), (**b**) reaction time (T = 80 °C, alcohol–oil molar ratio of 9:1, catalyst dosage of 6 wt%), (**c**) alcohol–oil molar ratio of the reaction (t = 3 h, T = 80 °C, catalyst dosage of 6 wt%), (**d**) catalyst dosage (t = 3 h, T = 80 °C, alcohol–oil molar ratio of 9:1).

The one-factor optimisation method was used, and Figure 5a shows the optimisation of the reaction temperature, in which it can be seen that the yield of biodiesel is 73.7% when the reaction temperature is 70 °C, and the yield esentially does not change much when the reaction temperature reaches 80 °C, 90 °C and 100 °C. This could be the result of higher temperatures leading to easier vaporisation of methanol, making little change in the conversion of biodiesel. So, the optimum reaction temperature was 80 °C. Figure 5b shows the optimization of the reaction time. At 1 h, 2 h and 3 h, the yield was gradually increased, and at 3 h it reached 82.2%, but at 4 h and 5 h the yield was 85.2% and 86.1%. For both, the increase is not much and both are essentially the same. The reason for this may be that the active sites of the catalyst were destroyed after too long a period of time so that the yield remained unchanged. The period of 3 h was determined to be the optimum reaction time in terms of time and cost. Figure 5c shows that biodiesel yield was determined at a reaction temperature of 80 °C, catalyst dosage of 6 wt%, reaction time from 3 h and alcohol–oil molar ratios of 3:1, 6:1, 9:1, 12:1, and 15:1, respectively. The molar ratio gradually increased. In the process, the biodiesel yield also gradually increased up to 89.6%, when the increase in the molar amount of methanol was equivalent to increase the concentration of the reactants, so that the reaction moved to the right, but too much methanol would lead to an increase in the resistance in the material convention, but also in order to save costs, so the molar ratio of 9:1 was the most suitable. Subsequent optimisations were performed under 9:1 conditions. Figure 5d shows the effect of the catalyst amount on the esterification reaction. The yield was gradually increased when the catalyst amount was gradually increased. The yield was already 85.0% at 6 wt% of catalyst and 86.6% at 8 wt% of catalyst, which was not a significant increase in biodiesel yield. The yield was 90.0% at 10 wt% of catalyst. Adding more catalyst would increase the yield to some extent, but not adding more catalyst is better as more catalyst will lead to higher costs and will also create some resistance to the conduction of the reaction. Combined with the above considerations, the optimum catalyst dosage was 6 wt%.

In an attempt to save the reaction time, save the cost of the reaction and increase the yield, the experimental conditions for the preparation of biodiesel were found to be 80 °C, the alcohol–oil molar ratio 9:1, the reaction time 3 h, and the catalyst dosage 6 wt%. UiO-66-SO_3_H catalysed the oleic acid esterification reaction under optimal reaction conditions to obtain a biodiesel yield of 85.0%, and UiO-66 catalysed the oleic acid esterification reaction under the same conditions to obtain a biodiesel yield of 20.5%.

### 2.4. Kinetic Study

The chemical formula for the UiO-66-SO_3_H catalysed esterification of oleic acid with methanol is given below:

$${\mathrm{CH}}_{3}{\left({\mathrm{CH}}_{2}\right)}_{7}\mathrm{CH}=\mathrm{CH}{\left({\mathrm{CH}}_{2}\right)}_{7}\mathrm{COOH}+{\mathrm{CH}}_{3}\mathrm{OH}\begin{array}{c} \mathrm{Catalyst}\\ ⇋ \end{array}{\;\mathrm{CH}}_{3}{\left({\mathrm{CH}}_{2}\right)}_{7}\mathrm{CH}=\mathrm{CH}{\left({\mathrm{CH}}_{2}\right)}_{7}{\mathrm{COOCH}}_{3}+H_{2}O$$

**Figure 6:**
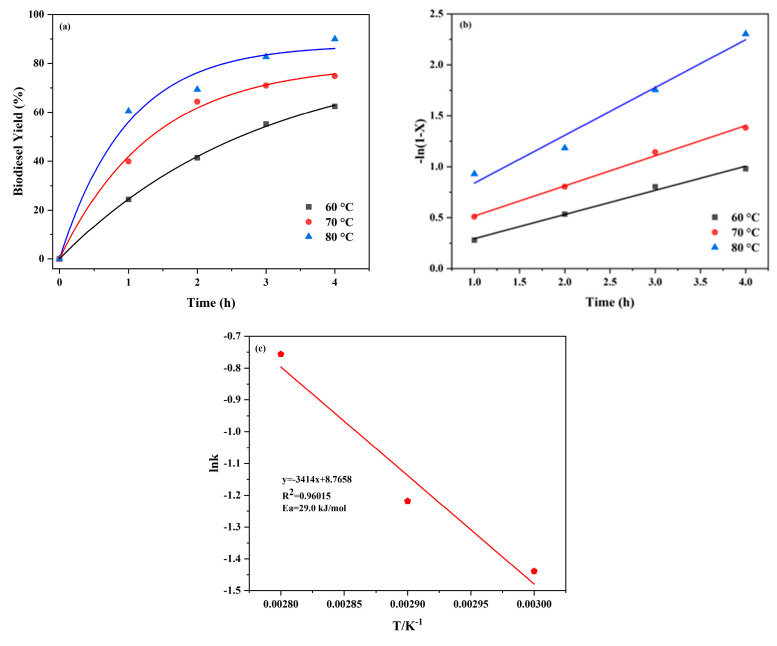
(**a**) Biodiesel reaction kinetics at different temperatures (333 K, 343 K, 353 K), (**b**) fitted plot of −ln(1 − x) with time and (**c**) linear fit of lnk /T.

Theoretically, the production of biodiesel requires a molar ratio of methanol to oil of 3:1. However, the above chemical equation is a reversible reaction between oleic acid and methanol, and in case the reaction proceeds in the positive direction, the amount of methanol has to be in excess. Figure 6a shows the determination of the biodiesel yield at different temperatures (333 K, 343 K, 353 K) for different times. On the graph it can be seen that the amount of methanol was much larger than the amount of oleic acid, and therefore, the excess amount of methanol present suppressed the secondary reaction and primarily drove the pseudo-primary kinetics [44,45]. In the absence of catalyst dosage, the yield of biodiesel was essentially zero, so the thermodynamic effects of the reaction were negligible and the resistance to mass transport during the esterification reaction was small or even negligible [46].

The rate expression can be formulated as Equation (2):

(2) $$-r=-\frac{dC_{A}}{dt}=kC_{A}$$

r denotes the rate of chemical reaction, C_A_ indicates the concentration in oleic acid, k rate constant and taking the natural logarithm of C_A_ gives the following Equation (3):

(3) $$-ln\left(1-X\right)=\mathrm{kt}$$

Combined with the Arrhenius equation, the activation energy can be calculated (4):

(4) $$\ln k=-\frac{E_{a}}{RT}+\ln A$$

X denotes the conversion rate of biodiesel, ln k is the logarithm of the rate constant, t denotes time, A denotes antecedent factor, Ea denotes activation energy and 1/T is the absolute temperature inverse.

The linear correlation plots of $-ln\left(1-X\right)$ versus time at different temperatures are shown in Figure 6b, presenting a good linear relationship. The reaction rate constant k and its corresponding R^2^ at different temperatures were obtained as shown in Table 2, indicating that the esterification process follows first-order kinetics. The activation energy Ea = 29.0 kJ/mol and A = 6.4 × 10^3^ min^−1^ was calculated from the slope of the lnk to 1/K fit line and the Arrhenius equation. The value of activation energy Ea was greater than 25.0 kJ/mol proving that the esterification of oleic acid catalysed by UiO-66-SO_3_H was kinetically controlled. The activation energy of this esterification reaction was also consistent with the range of activation energies reported in the literature for esterification reactions (17.9–51.9 kJ/mol) [47]. It can be seen in Table 3 that the activation energy of the reaction was relatively low in the present work, indicating that the catalyst (UiO-66-SO_3_H) significantly reduced the activation energy of the reaction, resulting in high catalytic activity.

### 2.5. Reusability Studies of Catalysts

**Figure 7:**
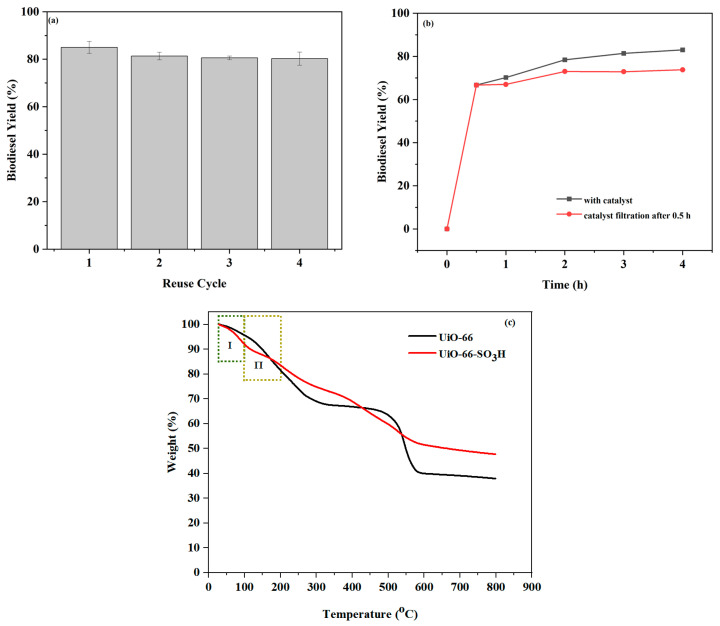
(**a**) Reusability study of UiO-66-SO_3_H catalyst. The reaction conditions were as follows: t = 3 h, T = 80 °C, catalyst dosage was 6 wt%, and the alcohol–oil molar ratio was 9:1. (**b**) Thermal filtration of UiO-66-SO_3_H (T = 80 °C, catalyst dosage was 6 wt%, and the alcohol–oil molar ratio was 9:1). (**c**) TGA mapping of UiO-66 and UiO-66-SO_3_H.

The reusability of catalysts can significantly decrease production costs and also reduce the impact on the environment. Thus, it is desirable to study the reusability of catalysts. In the course of each reaction cycle, the catalyst was centralised and washed several times with petroleum ether and ethanol to eliminate some impurities attached to the catalyst. The catalyst was dried in a hot oven at 50 °C for 5 h and then the reaction was performed again under the same experimental conditions as before. The reusability performance of UiO-66-SO_3_H is shown in Figure 7a. Four cycles were carried out, respectively. The third reusability yield was 80.6%, the fourth yield was 80.2% and the results of the times were not much different, which indicated that the catalyst has good reusability.

Thermal filtration is a conventional method of differentiating between catalyst types, and a comparison of biodiesel obtained after thermal filtration with no filtration allows for a quick determination of homogeneous/non-homogeneous solid catalyst types [40]. Typically, thermal filtration experiments of catalysts are measured under the same comparative experimental conditions. As shown in Figure 7b, it was demonstrated that removing the catalyst after 0.5 h, the biodiesel yield fluctuated little. Therefore, UiO-66-SO_3_H is a non-homogeneous phase solid catalyst with excellent catalytic performance.

The heat-stabilised TGA profiles of UiO-66 and UiO-66-SO_3_H are shown in Figure 7c. In the figure it can be seen that in the first stage of labelling the weight of UiO-66 decreased by 4.37% and the weight of UiO-66-SO_3_H decreased by 7.55%, which may be due to the high solvent ratio during the sulfonation process. In the second stage of labelling the weight of both the sulfonated and unsulfonated catalysts started to decrease rapidly, probably due to the removal of small molecules left in the pores by the catalysts. Therefore, this catalyst met the conditions for the catalytic preparation of biodiesel in this experiment, in which the maximum temperature for biodiesel production was 100 °C.

It can be seen from Table 4 that UiO-66-SO_3_H is an excellent catalyst. It has a short reaction time, suitable reaction temperature, good stability and reusable property. UiO-66-SO_3_H has good catalytic performance for biodiesel synthesised from high acid value feedstocks.

## 3. Materials and Methods

### 3.1. Materials

Anhydrous methanol (AR, 99.5%), petroleum ether (AR, >98.5%) and sodium chloride (AR, 99.5%) were purchased from Tianjin Zhiyuan Chemical Reagent Co., Ltd. (Tianjin, China). Anhydrous ethanol (AR, 99.7%) was purchased from Tianjin Fuyu Fine Chemical Co., Ltd.(Tianjin, China). N,N-Dimethylformamide (AR, 99.5%), Phenolphthalein (GR, 98%), Acetic Acid (AR, 99.5%), Zirconium Chloride (GR, 99%) and Sodium Hydroxide (GR, 97%) were supplied by McLean Biochemicals (Shanghai) Co., Ltd. (Shanghai, China). Oleic Acid (AR, 98%, AV = 185.9 mg KOH/g), Terephthalic Acid (AR, 99%) by Ron Reagent and Chlorosulfonic Acid (GR,99%) were purchased from Shanghai Xianding Bio-Technology Co., Ltd. (Shanghai, China). Potassium Hydroxide (GR, 85%) Aladdin and all chemicals can be used without further purification.

### 3.2. Preparation of Catalysts

UiO-66 was synthesised following the methodology of previous syntheses [40,52,53,54], by dissolving zirconium chloride (0.64 g, 2.7 mmol) and terephthalic acid (0.46 g, 2.8 mmol) in a reactor containing N, N-dimethylformamide (50.0 mL) and acetic acid (4.0 mL). The reaction was stirred, and the reaction was made for 24 h in a 120 °C hot oven. The reaction was centrifuged and strained to give a white solid, which was cleansed three times with anhydrous ethanol and N, N-dimethylformamide and dried in an oven at 50 °C. The product obtained was labelled as UiO-66.

### 3.3. Sulfonation of Catalysts

The sulfonation method was improved based on the previous literature [46]. UiO-66 (1.0 g) was added to a round-bottomed flask containing N, N-dimethylformamide (60 mL) and stirred at 35 °C. Chlorosulfonic acid (2.0 mL) was added and reacted for 2 h. White- coloured solid was filtered by centrifugation, rinsed with anhydrous ethanol and N, N-dimethylformamide, and the product derived from drying in an oven at 50 °C was assigned UiO-66-SO_3_H.

### 3.4. Characterisations

The crystal structure of the catalysts was analysed by X-ray diffraction (XRD, manufacturer Rigaku Japan, smartlab9). The external morphology of the samples was characterised by scanning an electron microscope (SEM, Thermo Fisher Scientific, APreo 2, USA). The specific surface area and pore volume of the samples were determined by N_2_ adsorption–desorption (BET, manufacturer Mack, USA, Micro for TriStar II Plus 3030). The chemical structure of the catalysts was analysed by Fourier transform infrared (FT-IR, Thermo Scientific, Nicolet-iS10). The thermal stability of the catalysts was determined by thermogravimetric analysis (Mettler TGA/DSC1) under nitrogen at 30 °C–800 °C with a temperature increase rate of 10 °C/min.

### 3.5. Acid Value Measurement

This was measured according torelevant literature [42]. Measure the volume ratio of 1:1 anhydrous ethanol and petroleum ether mixed solution in a 150 mL conical flask, add 3–4 drops of phenolphthalein indicator and titrate with 0.05 mol/L potassium hydroxide ethanol liquid to a slight red color and ensure that it does not fade within 30 s as the end point of titration. The volume of KOH-CH_3_CH_2_OH solution consumed is V. The titration was carried out using the regression titration method, followed by the rapid addition of the sample to be tested labelled m, and then the titration was carried out with an ethanol solution of potassium hydroxide.

The acid value (AV) was calculated using Equation (5):

(5) $$AV(mg\;KOH/g)=\frac{56.1\times V\times c}{m}$$

V-Volume of KOH-CH_3_CH_2_OH used in millilitres (mL)

c-Concentration of KOH-CH_3_CH_2_OH applied, in moles per litre (mol/L)

m-Mass of oleic acid in grams (g)

56.1—Molar mass of KOH expressed in grams per mole (g/mol)

### 3.6. Determination of Acid Density

A catalyst (40.0 mg) was added to an aqueous solution of 0.1 M NaCl (20 mL), with agitation at room temperature for 24 h. Then there is filtration of the liquid, inserting 3–4 drops of phenolphthalein indicator, using 0.01 mol/L NaOH of an aqueous solution to a slight red color that does not disappear within 30 s, which is the end point of the titration. Record the volume of consumed NaOH aqueous solution V.

Below is the acid density calculation Formula (6):

(6) $$H^{+}(\mathrm{mmol}/g)=cV/m$$

V—Volume of standard solution of NaOH in millilitres (mL)

c—Confluence of the standard solution of NaOH in moles per litre (mol/L).

m—Mass of the specimen in grams (g)

The acid density of UiO-66-SO_3_H was calculated to be 0.81 mmol/g based on acid-base determination.

### 3.7. Preparation of Biodiesel by UiO-66-SO_3_H Catalysed Hydrolysis of Oleic Acid

**Figure 8:**
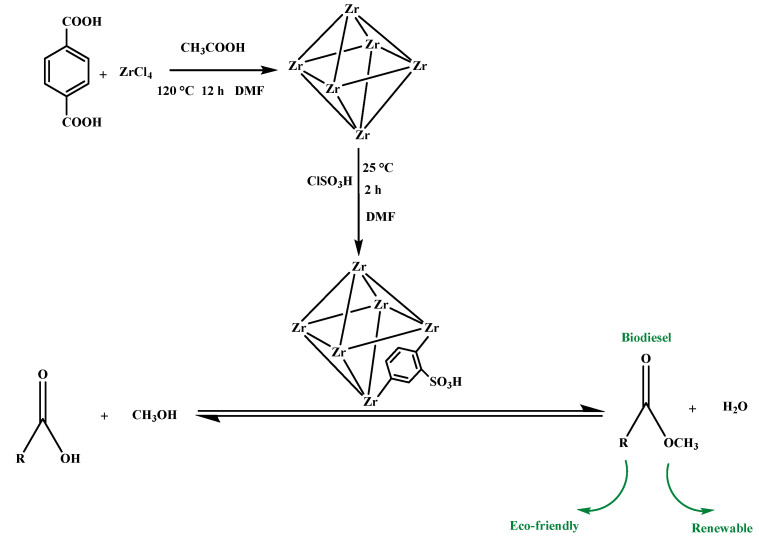
Biodiesel synthetic pathway diagram.

Oleic acid (1.0 g, 3.5 mmol, AV = 185.9 mg KOH/g) and anhydrous methanol (1.0 g, 31.5 mmol) together with 0.06 g of catalyst were combined in a 15 mL pressure-resistant flask in an oil bath with magnetic stirring (500 rpm) at 80 °C over a period of 3 h. Reactants were fully reacted and mixed with the catalyst. At the end of the reaction, the product was cooled to room temperature, the catalyst was recovered by centrifugal filtration, the clear liquid was taken and the excess water and methanol were removed by rotary evaporator, and the yield of biodiesel was analysed by nuclear magnetic resonance (NMR) [55]. The route of biodiesel preparation is shown in Figure 8.

### 3.8. Parameters Affecting Catalytic Activity

In an attempt to select the catalytic yield under optimal conditions and to save costs, the one-factor optimization method was used to optimise the experimental conditions. The biodiesel yields from methanol esterification with oleic acid were obtained at reaction temperatures of 60 °C, 70 °C, 80 °C, 90 °C and 100 °C and reaction times at 1 h, 2 h, 3 h, 4 h and 5 h. The molar ratios of methanol and oleic acid were 3:1, 6:1, 9:1, 12:1 and 15:1. The catalyst dosage of 2 wt%, 4 wt%, 6 wt%, 8 wt% and 10 wt% was obtained to obtain the biodiesel yield from the esterification of methanol with oleic acid.

### 3.9. Reaction Kinetics Studies

The kinetics towards UiO-66-SO_3_H catalysed by esterification by oleic acid were investigated by applying the controlled variable method. A molar ratio between methanol and oleic acid was 9:1, with reaction time being 1 h, 2 h, 3 h, 4 h and 5 h, and catalyst dosage being 4 wt%. The temperature of the transesterification reaction was 60 °C, 70 °C and 80 °C, respectively, and the biodiesel yield was calculated at the end of the reaction. The Arrhenius Equation (7) was used to evaluate the reaction kinetics of UiO-66-SO_3_H catalysed by esterification of oleic acid [43].

(7) $$k=Ae^{-E_{a}/\left(RT\right)}$$

## 4. Conclusions

In this paper, a straightforward method was taken to synthesise MOF (UiO-66-SO_3_H) materials with sulfonic acid groups. Successful grafting of the sulfonic acid moiety was demonstrated by systematic characterization, and the catalyst could serve as both Brønsted and Lewis acid active sites with a large specific surface area (913.8 m^2^/g, 502.4 m^2^/g). Under the reaction conditions optimised by the one-factor method, the yield of methyl oleate catalysed by UiO-66-SO_3_H could reach 85.0%. The kinetic study of the reaction showed that the reaction is a proposed primary reaction and has a low reaction activation energy (Ea = 29.0 kJ/mol), indicating that the UiO-66-SO_3_H catalyst has a high catalytic activity. The successfully synthesised solid acid catalysts used acetic acid as a moderator, which led to the formation of Brønsted and Lewis acid sites on the surface of the catalysts and greatly enhanced the catalytic activity. UiO-66-SO_3_H was subjected to reusability studies and showed good catalytic activity even after four repetitions. In conclusion, the synthesised non-homogeneous catalysts are of significance for the catalytic synthesis of efficient and green biodiesel.

## Figures and Tables

**Table 1:** Specific surface area and pore volume of different catalysts.

Sample	BET Surface Area (m^2^/g)	Pore Volume (cm³/g)	Average Particle Size (nm)
UiO-66-SO_3_H	913.8	0.09520	245.7
UiO-66	1226	0.1029	194.7

**Table 2:** Rate constants for the esterification of oleic acid catalysed by UiO-66-SO_3_H.

Temperature (°C)	Reaction Rate Constant (k)	Coefficient of Determination (R^2^)
60	0.2372	0.9928
70	0.2958	0.9958
80	0.4693	0.9759

**Table 3:** Comparison of activation energies of catalysts.

Catalysts	Activation Energy (KJ/mol)	Reference
UiO-66-SO_3_H	35.3	[46]
UiO-66/SA	41.5	[31]
Fe_3_O_4_@SiO_2_-SO_3_H	47.9	[45]
UiO-66-SO_3_H	29.0	This work

**Table 4:** Comparison of the performance of different catalysts.

NO.	Catalyst	Feedstock	Reaction Conditions	Biodiesel Yield (Conversion) (%)	Ref.
1	cotton stalk	Madhuca indica oil	T = 60 °C, CA = 5 wt%, M/O = 18:1, t = 5 h	89.2	[48]
2	HOP CLPS-SO_3_H-7	oleic acid	T = 60 °C, CA = 6 wt%, M/O = 10:1, t = 6 h	93.7	[49]
3	WP-SO_3_H-6	oleic acid	T = 100 °C, CA = 8 wt%, M/O = 20:1, t = 20 h	94.4	[50]
4	MnO_2_@Mn(btc)	oleic acid	T = 100 °C, CA = 3 wt%, M/O = 12:1, t = 12 h	98	[51]
5	UiO-66-SO_3_H	oleic acid	T = 80 °C, CA = 6 wt%, M/O = 9:1, t = 3 h	90.0	This study

CA—catalyst amount; T—reaction temperature; M/O—methanol to oil molar ratio; t—reaction time.

## Data Availability

Data are contained within the article.
